# Capture of Lipopolysaccharide (Endotoxin) by the Blood Clot: A Comparative Study

**DOI:** 10.1371/journal.pone.0080192

**Published:** 2013-11-25

**Authors:** Margaret T. Armstrong, Frederick R. Rickles, Peter B. Armstrong

**Affiliations:** 1 Marine Biological Laboratory, Woods Hole, Massachusetts, United States of America; 2 Department of Molecular and Cellular Biology, University of California Davis, Davis, California, United States of America; 3 Department of Medicine, School of Medicine, The George Washington University, Washington, DC, United States of America; Indian Institute of Science, India

## Abstract

In vertebrates and arthropods, blood clotting involves the establishment of a plug of aggregated thrombocytes (the cellular clot) and an extracellular fibrillar clot formed by the polymerization of the structural protein of the clot, which is fibrin in mammals, plasma lipoprotein in crustaceans, and coagulin in the horseshoe crab, *Limulus polyphemus*. Both elements of the clot function to staunch bleeding. Additionally, the extracellular clot functions as an agent of the innate immune system by providing a passive anti-microbial barrier and microbial entrapment device, which functions directly at the site of wounds to the integument. Here we show that, in addition to these passive functions in immunity, the plasma lipoprotein clot of lobster, the coagulin clot of *Limulus*, and both the platelet thrombus and the fibrin clot of mammals (human, mouse) operate to capture lipopolysaccharide (LPS, endotoxin). The lipid A core of LPS is the principal agent of gram-negative septicemia, which is responsible for more than 100,000 human deaths annually in the United States and is similarly toxic to arthropods. Quantification using the Limulus Amebocyte Lysate (LAL) test shows that clots capture significant quantities of LPS and fluorescent-labeled LPS can be seen by microscopy to decorate the clot fibrils. Thrombi generated in the living mouse accumulate LPS *in vivo*. It is suggested that capture of LPS released from gram-negative bacteria entrapped by the blood clot operates to protect against the disease that might be caused by its systemic dispersal.

## Introduction

Higher animals cope with invading pathogens by deploying a variety of defense systems that involve specialized immune defense cells and effector proteins and peptides that are found at the integumental surface and in the blood. The cellular and humoral components of the immune system function to neutralize and clear invading pathogens and foreign molecules introduced by pathogens, several of which are toxins or virulence factors. The fibrillar blood clot contributes to immunity in the arthropod, *Limulus polyphemus* (the American horseshoe crab)[1] and mammals[2]–[4] by immobilizing[2], [3] and killing[4] invading microbes and thereby preventing their dissemination throughout the internal milieu. The clot of *Limulus* is a polymer of the protein coagulin[5] and the clot of mammals is a polymer of fibrin[6]. The clot of the American lobster, *Homarus americanus*, the third animal in our study, is a polymer of a plasma protein, variously named clotting protein and very high density lipoprotein (VHDL). In all three species, these proteins polymerize into the fibrils of a robust extracellular matrix.

In addition to the passive entrapment of microbes invading at wound sites, the present report provides evidence for an active participation of the clot in immunity by its ability to capture and sequester the important microbial toxin, lipopolysaccharide (LPS, endotoxin). LPS is the principal lipid of the outer lamella of the outer membrane of Gram-negative bacteria[7] and is continuously shed into the environment by populations of these bacteria. Because lipid A, the central component of LPS, is a major agent of morbidity and mortality in humans and arthropods that experience infection by Gram-negative bacteria[8], [9], immunologists are interested in the systems for its inactivation and sequestration[10], [11]. We find that the extracellular fibrin clot and the platelet thrombus of the mammalian clotting system both bind LPS, as does the extracellular coagulin clot of the horseshoe crab, *Limulus polyphemus*, and the VHDL clot of the American lobster. It is suggested that the capture of LPS by the blood clot serves to reduce the dissemination of LPS released by Gram-negative bacteria that become entrapped in the clot following wounding of the integument, thus reducing the possibility that LPS from that source could cause systemic disease.

## Materials and Methods

### Materials

The following variants of LPS were used in these experiments: *Salmonella minnesota* R595 (Re) from List Biological Laboratories, Inc., cat # 304; *Escherichia coli* 011:B4 from Sigma-Aldrich, St Louis, MO cat # L2630 and F3665l (FITC conjugate); *Escherichia coli* 055:B5 from Sigma-Aldrich cat # L2880 and L2630 (FITC conjugate) and from Invitrogen Corporation, Carlsbad, CA, Cat # L-23351 (Alexa Fluor 488 conjugate); *Escherichia coli* O113:H10 from Associates of Cape Cod, Falmouth, MA, Cat # E0125 (Control standard endotoxin). Polymyxin B was from Sigma-Aldrich, Cat # P-1004 and Invitrogen Corp. cat # PI3236 (Oregon Green 514 conjugate). The Pyrotell LAL test reagent was from Associates of Cape Cod, cat # G5250. Human Research Grade Fibrinogen and Factor XIII were from Haematologic Technologies, Essex Junction, VT cat # HCI-0150R. Human plasma-derived thrombin was a generous gift from Dr. John W. Fenton (NY State Department of Health, Albany, NY). LPS-free human plasma-derived thrombin (800–1200 IU/ml) and fibrinogen (55–85 mg/ml) (Evithrombin and Human Fibrinogen/Evicel) were obtained from the Ethicon Division of Johnson & Johnson, NCK 63713-390-11, lot # Q51T030.

### Fibrin clot (mouse, human)

Human blood was drawn into 0.1 volumes of citrated saline (Sigma cat # C7165) and the blood cells and platelets were removed by centrifugation at 300×g×10 minutes and then the supernatant re-spun at 5,000×g×5 minutes. The citrated, platelet-depleted plasma (PDP) was stored in aliquots at −80°C. Human plasma was obtained from the corresponding author, Peter Armstrong, by the second author, Frederick Rickles, who is a physician, and is qualified to draw blood. Peter Armstrong gave his informed consent for the withdrawal of his blood for use in this investigation. This was conducted under IRB protocol # 2001-P-001867 issued by Harvard University to Professor Bruce Furie, Beth Israel Deaconess Medical Center, Boston, MA and the Marine Biological Laboratory, Woods Hole, MA. Mouse blood was aspirated into 0.1 volumes of citrated saline-dextrose from the vena cava of anesthetized mice and the blood cells were removed by centrifugation. Clotting was initiated by adding thrombin (1–4 U/mL-human) or Thromboplastin C Plus (Dade division, Baxter Scientific Products, Miami, FL-mouse) or by re-calcifying the plasma. The Beth Israel Deaconess Medical Center Institutional Animal Care and Use Committee approved all animal care and experimental procedures. Human clots were also prepared by allowing a drop of freshly-drawn blood to clot on a microscope coverglass, after which the cells were lysed by treatment with 0.5% Triton X-100 in Tris-buffered saline. The fibrin clot was also prepared by the thrombin-mediated proteolysis of purified human fibrinogen. A preparation of 0.5 mg/mL fibrinogen in 10 mM Ca^2+^, 0.15 M NaCl, 20 mM Tris, pH 7.3, with or without 5 µg/mL Factor XIII, was induced to clot by the addition of 1 U/mL thrombin (37°C, 1–3 h)[12]. The ε-amino groups of the arginine and lysine residues of the fibrin clot were derivatized, respectively, with phenylglyoxal [13] and trinitrobenzene sulfonate (TNBS) [14].

### Coagulin clot (Limulus)

Adult horseshoe crabs obtained from the Marine Biological Laboratory, Woods Hole, MA, were maintained in running seawater aquaria and fed thrice weekly with lobster muscle. After use, the animals were returned unharmed to the ocean. Horseshoe crab plasma was prepared as described previously[15]. A blood clot suitable for microscopic study was prepared as follows: two drops of hemolymph were collected by cardiac puncture into a 35 mm polystyrene Petri dish containing 1 mL pyrogen-free 3% NaCl (Travenol, Deerfield, IL, cat. # 2A1353) and incubated for 5 min at room temperature (T) to allow the blood cells to attach to the dish surface. The saline was then replaced with sterile-filtered *Limulus* plasma and incubated for 2–3 h at room T. During this period, the blood cells flatten on the dish surface, degranulate to release the proteins for clot formation, and establish a coagulin clot above the flattened cells.

### Plasma lipoprotein clot (American lobster)

Lobsters were maintained in individual cages in running sea water and were fed squid. After use, the lobsters were returned to the ocean unharmed. Hemolymph was removed from adult lobsters by aspiration from the heart and 30 µL droplets of fresh hemolymph were placed on the surface of plastic Petri dishes, where they soon clotted. Clots were incubated with LPS during or shortly after clotting or were extracted with detergent (0.5% Triton X-100 in 0.5 M NaCl, 10 mM CaCl_2_, 10 mM Tris, pH 7.3) and then incubated with LPS.

### Microscopy

FITC-LPS (*E. coli*, 055:B5, 3.9 µg FITC/mg LPS and *E. coli* 011:B4, Sigma, St Louis, MO) was suspended in Tris-buffered saline (0.5 M NaCl, 0.01 M Tris, pH 7.3 (lobster, *Limulus*), or 0.15 M NaCl, 0.01 M Tris, pH 7.3 (human, mouse)) then sonicated vigorously with a tip sonicator to reduce the size of the LPS micelles. Fibrin (human, mouse), coagulin (*Limulus*) and VLDL (lobster) clots on Petri dish surfaces were rinsed with saline, incubated with 0.4 mg/mL FITC-LPS for 1–2 h at room T, then the preparations were washed with saline and examined with the fluorescence microscope. The incubation and wash buffers sometimes contained elevated salt (1.0 M NaCl) or 2 M urea (Schwarz/Mann, Cambridge, MA, ultrapure; purged of cyanate ion by treatment with AG 501-X8 ion exchange resin, Bio-Rad, Richmond CA). Alternatively, clots were incubated with unlabeled LPS, washed, then incubated with biodipy- or Oregon Green 514-labeled polymyxin B (Invitrogen, Carlsbad, CA), a reagent that binds selectively to LPS[16], and examined with the fluorescence microscope. Control LPS-free clots treated with Oregon Green 514-polymyxin B failed to stain.

### Intravital microscopy of the murine thrombus

Intravital videomicroscopy of the cremaster muscle arterial circulation of wild-type C57BL/6J mice was performed as described by Falati *et al.* [17]. The Beth Israel Deaconess Medical Center Institutional Animal Care and Use Committee approved all animal care and experimental procedures. Mice were pre-anesthetized with ketamine, xylazine, and atropine and were cannulated via the jugular vein. The cremaster muscle was exteriorized onto a transparent observation platform and kept moist with bicarbonate-buffered isotonic saline maintained at 36°C. Nembutal for maintenance of anesthesia, AlexaFluor 647-labeled rat anti-mouse CD41 antibody (Fab fragment, clone MWReg30, Emfret Analytics, Eibelstadt, Germany) for immunostaining of the platelet thrombus, and AlexaFluor 488-labeled LPS (E. coli serotype 055:B5, molar labeling index 0.46, Invitrogen) were introduced into the circulation via the jugular cannula. At the completion of the experiment, the mouse was euthanized with a lethal dose of Nembutal administered through the jugular cannula. Each of two mice received 5 µg of LPS delivered in 100 µL of saline. Direct observation of arteries servicing the cremaster muscle was conducted with an Olympus AX microscope with ×40 0.6 and ×60 0.9 water-immersion objectives[18].

To provoke thrombus formation, the vessel wall of the artery was subjected to injury with the beam of a Micropoint Laser (Photonics Instrument) focused through the microscope objective. Digital images of the resulting thrombus were captured for transmitted visible light, and at the emission maxima for the AlexaFluor 488 and 647 reporter dyes to document the dynamics of thrombus formation, as previously described[17] for the capture of AlexaFluor 488-labeled LPS in the thrombus. Control sequences of laser-provoked thrombi were collected at these two wave lengths prior to introduction of the AlexaFluor 488-labeled LPS, then the labeled LPS was introduced into the system and additional thrombi were provoked and filmed.

### Image analysis

Image analysis used Slidebook (Intelligent Imaging Innovations). Images were captured digitally at up to 50 frames/sec. The analysis program assigned an intensity value between 0 and 4095 for each pixel of each frame. For display that value is converted to pseudo-color of associated intensity for the 488 (green) and 647 (red) channels. Background values were established in a rectangle positioned upstream of the thrombus and prior to its formation. This value was subtracted from each pixel to yield the thrombus-specific fluorescent intensities for each pixel. The analysis delivered colored frames that combined sequential transmitted bright-field, and fluorescent images in the green and red channels and a graphical summary of the variation with time of the specific fluorescence intensity for the entire image in green and red channels.

### LAL assay for LPS

The Pyrotell LAL (Limulus amebocyte lysate) kit (Associates of Cape Cod, Falmouth, MA, cat # G5250) was used to confirm the absence of exogenous endotoxin (LPS) in the reagents and to quantify the capture of LPS by the blood clot of the horseshoe crab, lobster, and human using the manufacturer's instructions. LPS (*E. coli* 0113:H10, “Control Standard Endotoxin”, cat E0125, Associates of Cape Cod) dissolved at the recommended concentration of 25 µg/mL in pyrogen-free distilled water with extensive sonication was added to freshly drawn blood and the blood was incubated for a time sufficient to allow for clotting and clot contraction to occur. Samples of the resulting serum were diluted 1∶10–1∶1000 in pyrogen-free water and the concentration of LPS that remained in the serum was quantified by coagulation of the LAL reagent. Prior to being subject to the LAL assay, human serum was diluted 1∶10 and incubated at 70°C for 10 min to inactivate endogenous inhibitors of the LAL test[19], [20], principally α_2_-macroglobulin[21]. In our hands, this assay was able to detect 0.1 ng/mL of LPS diluted in saline. The LPS capture efficiency of the clot was estimated by determining the maximum quantity of LPS that could be removed from whole blood during the formation of the blood clot. Controls included the determination of LPS concentrations in plasma and serum prepared at the same time from the same animal.

### AlexaFluor 488-labeled liposomes

A 6∶1 mixture of phosphatidylcholine: phosphatidylethanolamine (Avanti Polar Lipids, Alabaster AL) was dissolved in anhydrous chloroform and the chloroform evaporated with a stream of nitrogen. The dried lipid mixture was suspended in 0.1 M NaHCO_3_ containing 0.1 mg/mL AlexaFluor 488 5-TFP (Invitrogen) and sonicated until clear. After incubation overnight at 4°C, the preparation was separated from unconjugated dye by dialysis.

## Results

Capture of LPS by the blood clot was quantified using the Pyrotell version of the LAL (Limulus Amebocyte Lysate) test, which utilizes LPS-elicited coagulation of the LAL reagent to quantify the concentration of LPS in a given sample. The assay was routinely able to detect 0.1 ng/mL of LPS added to pyrogen-free water. The *Limulus* blood clot was able to reduce the concentration of LPS free in solution to below 10 ng/mL for initial concentrations of LPS as high as 3 µg/mL (Table 1). Although horseshoe crab plasma contains a form of C-reactive protein that binds LPS[22] and large quantities of protease inhibitors that might interfere with the proteases involved with the LAL test[23], the presence of *Limulus* plasma did not affect this detection limit, indicating that plasma did not contain inhibitors of the LAL test. Blood clotting in the horseshoe crab is initiated by exocytosis of the blood cells to release coagulogen, the protein that is cleaved to develop the coagulin clot, and to initiate blood cell aggregation to form the cellular thrombus[24]. Exocytosis releases a suite of LPS-binding proteins as well, which include anti-LPS factor (ALF), a cationic protein that binds and detoxifies LPS[25] and Factor C, a LPS-activated protease[26]. These soluble proteins make no apparent contribution to removal of LPS during clotting nor do they interfere with the LAL test - serum produced by exposure of whole blood to the calcium ionophore A23187 (10 µM), which provokes the same exocytosis reaction as does LPS[27] and could be presumed to result in the same release of soluble LPS-binding proteins from the secretory granules of the blood cells, fails to reduce the sensitivity of the LAL test (data not shown). Thus, essentially all of the LPS removed during clotting of whole blood of the horseshoe crab is removed by the blood clot. The blood clot of the lobster showed a measurable, but less effective ability to capture LPS than did the *Limulus* clot (Table 2).

**Table 1:** Capture of LPS by the *Limulus* blood clot^1^.

Initial LPS in the blood (ng/mL)	Dilution prior to assay	LAL coagulation	Maximum concentration of LPS in the serum (ng/mL)	Minimum LPS capture per 1 mL blood (ng)
100	10	No	1	99
400	10	No	1	399
1000	10	Yes		
1000	100	No	10	990
3000	100	No	10	2990

1 LPS from *E. coli* serotype O113:H10 was added to freshly drawn *Limulus* hemolymph and the preparation was incubated to allow the blood to clot and the clot to retract. The serum was diluted with pyrogen-free deionized water and assayed for residual LPS using the Pyrotell assay, in which a positive result is shown by coagulation of the LAL test reagent (column 3). The assay was sensitive to 0.1 ng/mL LPS both in water and in *Limulus* plasma (data not shown). Dilution of the samples elevated the minimum detectible concentration of LPS to the values shown in column 4 of the table. The amount of LPS removed from solution by the clot formed from 1 mL of whole blood is shown in column 5. Selected data points from this individual trial were replicated in 3 additional trials.

**Table 2:** Capture of LPS by the lobster blood clot^1^.

Initial LPS in the blood (ng/mL)	Dilution prior to assay	LAL coagulation	Maximum concentration of LPS in the serum (ng/mL)	Minimum LPS capture per 1 mL blood (ng)
100	100	Yes		
100	100	Yes		
100	150	No	15	85
100	200	No	20	80
100	200	No	20	80
100	400	No	40	60
100	500	No	50	50
800	1000	Yes		
800	1333	Yes		
800	2000	No	200	600

1 LPS was added to freshly collected lobster blood at the concentrations indicated in column 1, then the blood was allowed to clot. After a 1 h incubation at 23°C, the serum was collected and diluted in LPS-free water as indicated in column 2 and assayed by the LAL test (column 3), as described in Materials and Methods. The sensitivity of the assay when LPS was added directly to cell-free lobster hemolymph was 0.1 ng/mL LPS, but due to the dilution of the experimental samples, the detection limit of the maximum concentration of free LPS remaining in solution in the serum was correspondingly elevated, as indicated in column 4. Subtraction of maximum free concentration of LPS from the initial concentration yields the minimum amount of LPS captured by the clot produced by 1 mL of lobster blood (column 5). Selected data points from this individual trial were replicated in 1 additional trial.

The clot produced *in vitro* by whole human blood has been reported to capture approximately 1 µg of LPS per mL of blood[28]. The mammalian clot has two elements - the extracellular fibrin clot and the cellular thrombus, the latter of which is composed principally of aggregated blood platelets[6]. Capture of LPS appears to be largely a function of the platelet thrombus/fibrin clot because we found a similar capture efficiency for the clots produced by platelet-rich plasma, which was depleted of red cells and nucleated blood cells (Table 3) with the fibrin clot being responsible for the capture of most of the LPS in this *in vitro* system (Table 4).

**Table 3:** Capture of LPS by the complete human blood clot (platelet thrombus and fibrin clot)^1^.

Initial LPS in the blood (ng/mL)	Dilution prior to assay	LAL coagulation	Maximum concentration of LPS in the serum (ng/mL)	Minimum LPS capture per thrombus of 1 mL of platelet-rich plasma (ng)
10	10	No	1	9
100	10	Yes		
100	50	No	5	95
200	50	No	5	195
200	100	No	10	190
400	100	No	10	390
1000	250	Yes		
1000	500	No	50	950

1 LPS was added to a suspension of platelet-rich plasma at the concentrations indicated in column 1, after which the suspension was induced to clot by the addition of LPS-free recombinant thrombin. After a 1 h incubation at 37°C, the clot was removed and the serum was diluted in LPS-free water as indicated in column 2, heated for 10 min at 70°C to inactivate endogenous inhibitors of the LAL test (presumably α_2_-macroglobulin), and assayed by the LAL test (column 3), as described in Materials and Methods. The sensitivity of the assay was 0.1 ng/mL LPS, but due to the dilution of the experimental samples, the maximum concentration of free LPS remaining in solution in the serum was correspondingly elevated, as indicated in column 4. Subtraction of the maximum free concentration of LPS from the initial concentration yields the minimum amount of LPS captured by 1 mL of platelet-rich plasma (column 5). This is our sole trial for this condition and serves as a replicate for data reported previously by Levin et al [28].

**Table 4:** Capture of LPS by the human fibrin clot^1^.

Initial LPS in the blood (ng/mL)	Dilution prior to assay	LAL coagulation	Maximum concentration of LPS in the serum (ng/mL)	Minimum LPS capture per fibrin clot of 1 mL of platelet-depleted plasma
10	10	No	1	9
100	10	Yes		
100	10	Yes		
100	50	No	5	95
200	50	No	5	195
200	100	No	10	190
400	100	No	10	390
400	100	No	10	390
400	100	Yes		
1000	250	No	25	975
1000	500	No	50	950

1 LPS was added to a suspension of platelet-depleted plasma at the concentrations indicated in column 1, then the suspension was induced to clot by the addition of LPS-free recombinant thrombin. This is plasma from the same sample of blood used for Table 3, after removal of the platelets by centrifugation with a microcentrifuge at top speed. After a 1 h incubation at 37°C, the fibrin clot was removed and the serum was diluted in LPS-free water as indicated in column 2, heated for 10 min at 70°C, and assayed by the LAL test (column 3), as described in Materials and Methods. The sensitivity of the assay was 0.1 ng/mL LPS, but due to the dilution of the experimental samples, the maximum concentration of free LPS remaining in solution in the serum was correspondingly elevated, as indicated in column 4. Subtraction of maximum free concentration of LPS from the initial concentration yields the minimum amount of LPS captured by 1 mL of platelet-rich plasma (column 5). Selected data points from this individual trial were replicated in 7 additional trials.

**Figure 1:**
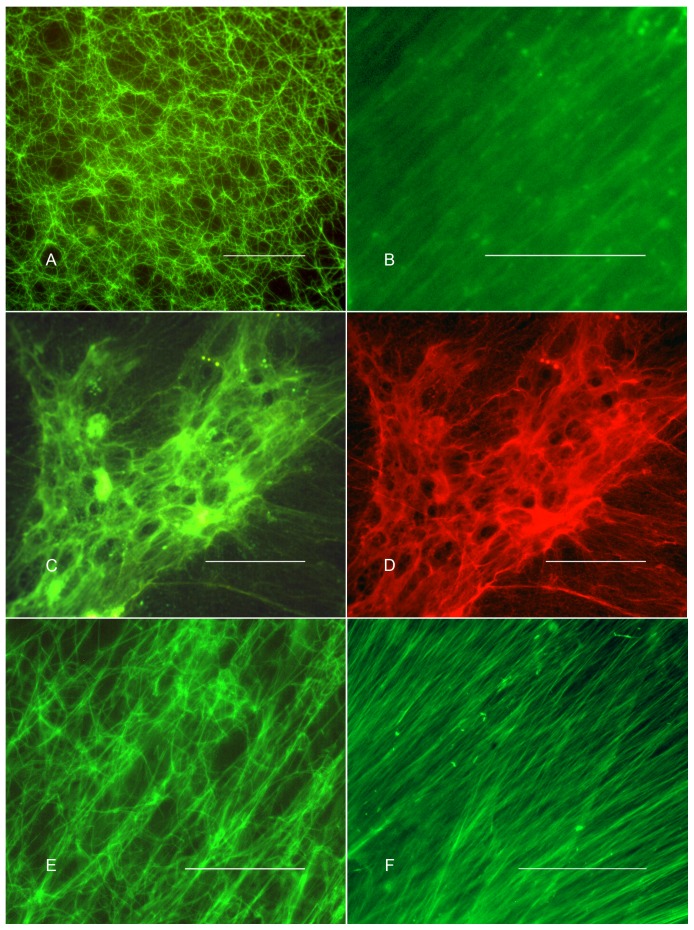
Decoration of the extracellular blood clot by lipopolysaccharide (LPS). FITC-LPS (*E. coli* O55:B5) decorates the fibrin fibrils of the human clot prepared from platelet-depleted plasma (Fig 1A), the plasma lipoprotein clot of the lobster (Fig 1B), and the coagulin fibrils of the *Limulus* clot (Fig. 1C). The *Limulus* clot shown in Fig 1C was also immunostained with a rabbit anti-coagulin antibody and DyLight 549 Goat anti-rabbit whole IgG second antibody to show the location of the coagulin fibrils of the blood clot. Figure 1D shows the same field of the *Limulus* blood clot as is shown in Figure 1C but illuminated with the rhodamine filter set to show the DyLight 549 signal, demonstrating the co-localization of LPS and the coagulin structural protein of the clot. The lipid A core of LPS is sufficient for binding to the fibrin clot because a form of LPS that lacks carbohydrate, LPS of *S. minnesota* R595 (Re), also binds to the fibrin clot (Fig. 1E). Here, biodipy-conjugated polymyxin B, a LPS-binding probe, was used as the fluorescent reporter for the localization of LPS. Fibrin clots prepared by thrombin-mediated clotting of pure human fibrinogen (Hematologic Technologies) also decorate with FITC-LPS (*E. coli* O55:B5) (Fig. 1F). The micrographs were produced with a Zeiss Axioimager microscope with a 40/0.75 objective. These representative micrographs are drawn from as many as 10 replicates (Fig. 1A) to as few as 2 replicates (Fig. 1B). Scale bar, 50 µm.

Fluoresceinated LPS decorated the fibrils of the mammalian, lobster, and *Limulus* blood clots as seen by microscopic examination (Figure 1A–D). Human clots prepared from PDP (Fig. 1A) or from whole blood (not shown) were decorated with FITC-LPS. Decoration of the fibrin clot by LPS was inhibited in the presence of a ten-fold molar excess of polymyxin B, an LPS-binding molecule (not shown). At least two possibilities exist by which LPS binding is mediated - either LPS binds directly to the structural protein of the clot (fibrin, VHDL, or coagulin) or LPS binds to one or more “adaptor” proteins from the plasma or hemolymph that themselves become linked to the clot, where they then bind LPS. A variety of proteins from plasma or hemolymph are known to decorate the fibrin and coagulin clots[29], [30] and one or more of these might be an adaptor protein responsible for LPS binding to the structural elements of the clot. Instead, binding to the mammalian clot appears to result from a direct association of LPS with fibrin. Depletion of plasma of LPS-binding proteins by exposure to LPS-Sepharose prior to the initiation of clotting failed to affect the subsequent binding of LPS to the clot. Furthermore, FITC-LPS decorates clots prepared from purified fibrinogen (free of contaminating proteins) that was induced to polymerize by thrombin-catalyzed proteolytic cleavage (Fig. 1F).

The difficulties of quantifying the fluorescence images due to the indeterminate thickness of the fibrin fibrils and the difficulty of working at high concentrations of LPS have frustrated the investigation of competitive inhibition of FITC-LPS binding by unlabeled LPS. Binding to the mammalian fibrin and *Limulus* coagulin clots was not noticeably affected by high salt (1.0 M NaCl) or 2 M urea (not shown), suggesting that binding is relatively strong. Binding of LPS to both clots appears to be selective because AlexaFluor 488-conjugated liposomes composed of a 6∶1 ratio of phosphaticylcholine:phosphatidylethanolamine, as a control for non-specific lipid interaction with the clots, failed to decorate either type of clot (not shown).

**Figure 2:**
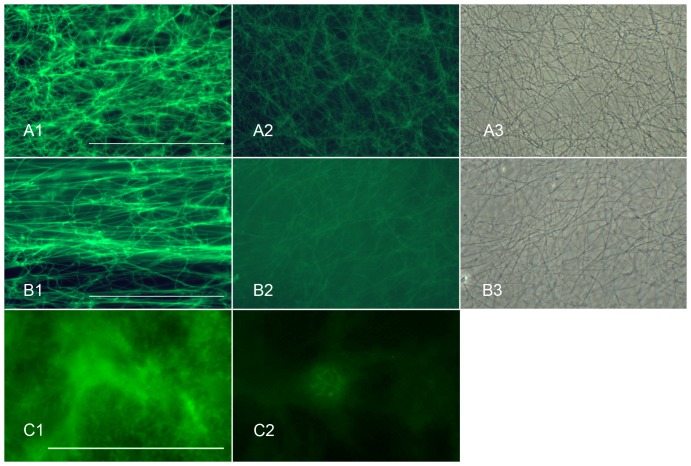
Derivitization of the ε-amino groups of lysine and arginine reduces capture of LPS by the blood clot. Treatment of the fibrin clot with TNBS to derivitize the ε-amino groups of lysine reduces capture of FITC-LPS (Fig. A1, untreated control fibrin clot, fluorescent LPS; Fig. A2, TNBS-treated fibrin clot, fluorescent LPS; Fig A3, same field as Fig A2, phase contrast). Similarly, treatment of the fibrin clot with PGO to derivitize the ε-amino groups of arginine (Fig. B1, untreated control fibrin clot, fluorescent LPS; Fig B2, PGO-treated fibrin clot, fluorescent LPS; Fig. B3, same field as Fig B2, phase contrast) also reduces LPS capture by the clot fibrils. Treatment of the coagulin clot of Limulus with PGO reduces capture of LPS by the clot (Fig. C1, untreated control coagulin clot; Fig. C2, PGO-treated coagulin clot). The PGO-treated specimen shows faint fluorescence of the nuclei of the underlying blood cells but no fluorescence of the overlying coagulin clot. TNBS treatment causes cytolysis of the *Limulus* blood cells and separation of the coagulin clot from the petri dish surface, preventing assessment of its effects on LPS capture in this system.

LPS consists of three covalently-linked elements, the lipid A moiety with six or seven hydrocarbon chains attached to a phosphorylated glucosamine disaccharide head group, a core oligosaccharide bound to the lipid A moiety via keto-deoxyoctanate (KDO) residues, and the O-antigen polysaccharide chain, which can be hundreds of monosaccharide residues long in some variants of LPS[7]. Binding of LPS to the fibrin clot does not appear to require the core oligosaccharide or the O-antigen carbohydrate chain because a form of LPS that lacks these constituents, LPS from *Streptococcus minnesota* R595 (Re), bound to the fibrin clot (Fig. 1E). Because basic amino acids feature importantly in the well-characterized binding motifs of LPS-binding proteins [31], [32], it is of interest to determine if arginine and lysine play a role in the capture of LPS by the blood clot. Indeed derivitization of the ε-amino group of the lysine residues of the fibrin clot with TNBS (Figure 2a) and arginine residues of the fibrin (Figure 2b) and coagulin (figure 2c) clots with phenylglyoxal did reduce capture.

**Figure 3:**
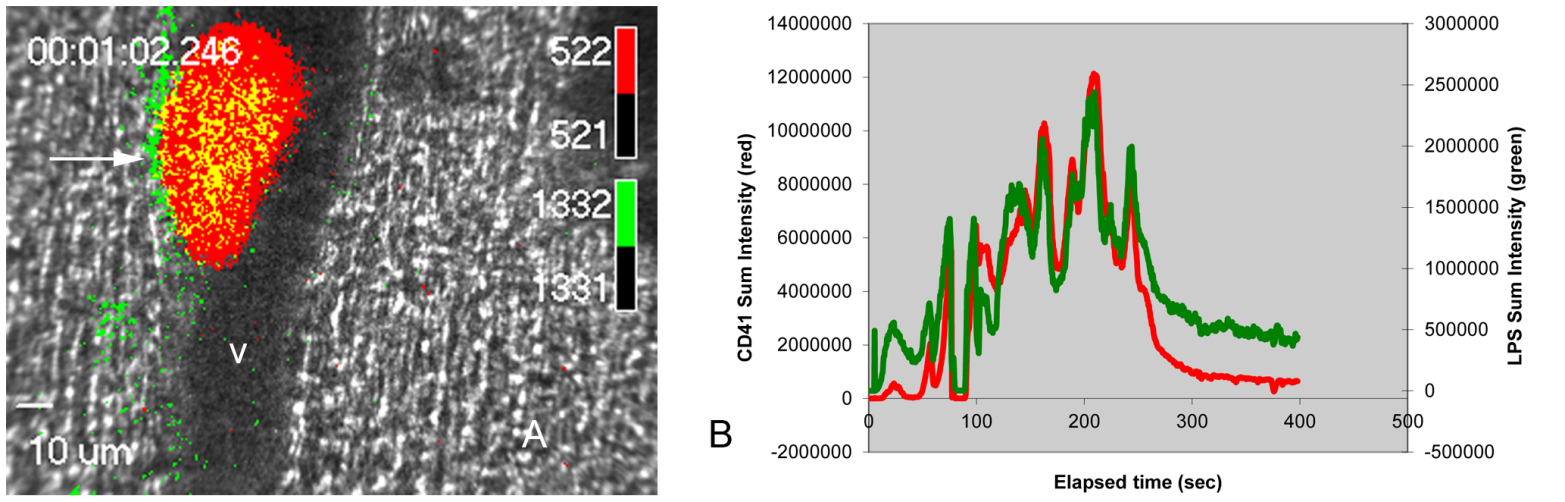
Capture of LPS *in vivo* (*E. coli* O55:B5) by the platelet thrombus. Intravital clots produced in large arteries of the living mouse cremaster muscle were challenged with AlexaFluor 488-labeled LPS introduced into the circulatory system. Figure 3A: the platelet thrombus is marked with AlexaFluor 647-labeled anti-CD41 Fab monoclonal antibody (red signal) and the location of LPS binding by AlexaFluor 488-labeled LPS (green signal). Where the location of the platelet thrombus coincides with LPS, the signal is yellow. In addition to binding to the thrombus, LPS is found at the interface of thrombus and blood vessel wall (arrow, green signal), which is the predominant location of the fibrin clot outside of the bounds of the platelet thrombus. The direction of blood flow in the artery is from the top of the figure to the bottom. v, lumen of the blood vessel. Figure 3B: time course of association of AlexaFluor 488-labeled LPS (*E. coli* O55:B5) with the site of the laser-provoked thrombus *in vivo*. Following laser damage to the blood vessel wall of the artery of the cremaster muscle of the mouse, the thrombus enlarges gradually as platelets accumulate at the damage site. The thrombus then shrinks precipitously as fragments of the thrombus break free and are carried away by the flowing blood. This is reflected in the 647 signal of the AlexaFluor-647-labeled anti-CD41 antibody (red trace). The association of LPS with the thrombus, measured by the 488 signal (green trace), shows a close correlation with the volume of the thrombus. These experiments were replicated in 17 thrombi from 2 mice.

To determine if LPS capture by the clot can occur *in vivo*, we analyzed video records produced by intravital microscopy of the thrombus developed by laser-elicited damage to the vascular endothelium of arteries perfusing the cremaster muscle of the living mouse. The mice were infused with AlexaFluor 488-labeled LPS introduced into the blood. In this system, the platelet thrombus that forms at the locus of a laser-induced injury of the blood vessel wall was identified by the adherence of platelets labeled with an AlexaFluor 647-labeled antibody against CD41, a cell surface protein of the blood platelet. The green label of LPS was found to be co-localized with the red label of the platelet thrombus (yellow pixels, Fig. 3A) and was seen at the thrombus-vessel wall interface and extending beyond the bounds of the thrombus (arrow, Fig. 3A). This is a location of the fibrin clot external to the bounds of the platelet thrombus[33] and the green signal is consistent with the possibility that, in addition to the platelet thrombus, the labeled LPS has bound to the fibrin clot that forms at the juncture of thrombus and blood vessel wall. A fibrin clot also is present within the platelet thrombus[17] and it is not possible to determine if the majority of the yellow signal, where the green and red fluorophores co-localize, is due to direct binding of the fluoresceinated LPS to platelets or to fibrin contained within the platelet thrombus. These clots are dynamic, with episodes of expansion as platelets accumulate at the injury site and abrupt events of retraction, when portions of the thrombus break free and are carried away with the flowing blood (video S1). The intensity of the 488 nm signal, a measure of the amount of LPS associated with the clot, shows close temporal correlation with the 647 nm signal, a measure of the volume of the thrombus (Fig 3B).

## Discussion

An important role for the blood clot in immune defense is suggested by a variety of observations. The mammalian and *Limulus* clots capture microbes both by entrapment within the fabric of the clot[3], [9] and by adhesive capture at the surface of the clot[1]. The mammalian clot can kill some pathogenic bacteria[4]. These processes function to immobilize and destroy microbes at the wound site, impeding their ability to cause systemic disease. Indirect evidence for a role of the clot in immunity is suggested by the presence of fibrinolytic protease systems as essential virulence factors for a broad variety of microbial, protozoan, and metazoan parasites[34], [35], suggesting that destruction of the clot is essential, in these instances, for pathogen virulence. Microbes can activate the exocytosis of the proteins of the clotting pathway from secretory granules of the blood cells[27], [36] and coagulation of the coagulin clot[5] in *Limulus* and can activate the clotting pathway of fibrinogen in mammals[37], the latter generally thought to be mediated principally by interaction of LPS with the “contact factors” of clotting (factors XII, XI, high-molecular-weight kininogen and kallikrein)[38]. Additionally, activation of mammalian[39], [40] and *Limulus* [41], [42] clotting systems can trigger in parallel a variety of other arms of the innate immune system.

The lipid A component of LPS is the causative agent of many of the pathological reactions observed in patients with gram-negative sepsis, a potentially lethal condition that claims more than 100,000 lives annually in the USA[8]. Horseshoe crabs are also susceptible to the introduction of LPS into the hemocoel, with death resulting from a disseminated activation of clotting and an arrest of the circulation of the hemolymph[9], [43], [44]. Probably because LPS is such a potent toxin, long-lived animals, as exemplified by humans, lobster, and *Limulus*, have multiple systems for recognition and detoxification of LPS. Activation of the canonical LPS signaling pathway in mammals involves the binding of LPS to lipopolysaccharide-binding protein (LBP) and CD14 and the binding of that complex to an MD-2-toll-like receptor 4 (TLR4) complex at the surfaces of phagocytes[45]–[47]. Capture by the murine platelet thrombus may involve binding of LPS to platelet TLR4 and CD62 at the surfaces of platelets[46], [47]. Several other discrete pathways for clearance and detoxification of LPS have been identified in mammals[10], [11]. In the horseshoe crab, factor C mediates the best-characterized LPS recognition system. Factor C is an LPS-activated serine protease, which operates as a pleiotropic LPS-binding agent. Factor C activation is the initial step in the clotting cascade in *Limulus* [26], mediates the binding of LPS to the surfaces of horseshoe crab hemocytes[48], and promotes hemocyte exocytosis. The latter event leads to the release of coagulogen and the proteases of the clotting cascade from the hemocytes[49]. The best characterized LPS-detoxifying agent in the horseshoe crab is ALF, a cationic protein from the hemocytes that binds and detoxifies LPS[25]; ALF is a candidate for therapeutic use in the management of gram-negative sepsis in humans[50].

The capture of LPS by the mammalian platelet/fibrin thrombus, by fibrin, VLDL, and coagulin clots of mammals, lobsters, and horseshoe crabs, respectively, represents a novel LPS sequestration pathway. Figure 1 shows that capture involves the direct binding of LPS to the surfaces of the clot fibrils rather than the physical sequestration of soluble LPS into fluid compartments of the clot. It is possible that LPS capture by the clot reduces the concentration of LPS free in solution both in situations where the clot is active at wounds to the integument and also for intravascular clots. Both in humans[51] and in the horseshoe crab[52], disseminated intravascular coagulation (DIC) is an important feature of the pathology of Gram-negative sepsis and is thought to contribute to the generalized organ failure that characterizes the terminal stages of the disease in humans by inducing small vessel thrombosis. Our demonstration of capture of LPS by the intravascular clot in the mouse suggests that the intravascular blood clot may reduce the concentration of circulating LPS during this process, but one assumes that only in the milder forms of Gram-negative sepsis would this be expected to ameliorate the severity of pathophysiology of DIC. A variety of therapeutic approaches to sepsis by manipulation of mammalian blood clotting pathways (e.g. treatment of DIC) have not yet proved dramatically successful at reducing mortality)[53]. It is possible that improving the efficacy of binding of LPS to early thrombus formation prior to the onset of DIC might be a rational approach.

The binding to the mammalian fibrin clot, rather than requiring an adaptor protein associated with the clot, appears to operate by direct binding to fibrin. The binding does not appear to require lectin-type interaction with carbohydrates of the O-polysaccharide of LPS, since forms of LPS lacking this moiety bind perfectly well to fibrin clots. The binding of LPS to fibrin and coagulin clots does not appear to be due to “non-specific” lipid interaction, since neither type of clot binds liposomes composed of the conventional membrane-type phospholipids, phosphatidylcholine:phosphatidylethanolamine. However, LPS is acknowledged to be a “sticky” molecule that attaches to solid surfaces, presumably by ionic and/or hydrophobic bonds. The best-characterized LPS binding motif of LPS-binding proteins includes a quartet of closely apposed lysine and arginine residues whose ε-amino groups interact with the negatively-charged phosphates of the phospho-glucosamine residues of the lipid A backbone [31], [54], [55]. Derivatization of the ε-amino groups of arginine by treatment of the fibrin (Fig. 2a) and coagulin (Fig. 2b) clots with phenylglyoxal and derivatization of the ε-amino acids of lysine of the fibrin clot by treatment with TNBS (Fig. 2c) reduced capture of fluoresceinated LPS, consistent with the notion that both arginine and lysine residues of the clot are important for LPS capture.

The binding of LPS to the fibrin, coagulin, and VLDL clots is relatively strong, resisting treatment with high salt. The maximum LPS capture capacity of the clots generated by 1 mL of blood is approximately 3 µg for the horseshoe crab (Table 1) and 1 µg for human (Table 3). Since each bacterial cell has approximately 3.5×10^6^ molecules of LPS or an amount of approximately 60 fg[56], 1 µg of LPS would represent the entire LPS content of 1.67×10^7^ bacterial cells.

Although the capacity of fibrin and VHDL clots to capture LPS is relatively high, the avidity is surprisingly low, as demonstrated by the significant quantities of LPS remaining in serum after clotting (Tables I–IV). Any attempt to assign molar binding parameters to this situation are complicated by the variability of the molecular mass of monomeric LPS due to the variability of the size of the O-polysaccharide chain even in preparations of LPS of a given serotype. A more significant contribution to variability derives from the tendency of LPS, an amphipathic molecule, to exist in solution in higher order aggregates of variable size[57]. Further complicating a quantitative interpretation of the relative contribution of this system to innate immunity is the association of LPS with various LPS-binding proteins of the plasma[11], which may affect association with the clot and/or affect the multimeric aggregation state of LPS. Indeed, if a majority of the LPS in the system is multimeric, then the molar concentration of the LPS micelles would be far lower than that estimated by dividing the nominal concentration of LPS by the molecular weight of the LPS monomer, with a corresponding effect on any estimate of molar binding parameters.

In summary, we identify here a newly recognized system for the sequestration of the important microbial toxin, lipopolysaccharide. Binding to the clot is suggested to be important for the capture and sequestration of LPS released by microbes entrapped in the clot during their entry, for example, through wounds in the integuments. As such, sequestration of LPS provides a mechanism for reducing the impact that would follow from the release of that LPS into the systemic circulation.

## Supporting Information

Video S1 **Time-lapse video of the capture of AlexaFluor 488-labeled LPS by the intravital clot.** AlexaFluor 488-labeled LPS (pseudocolored green) introduced into the circulation of a living mouse is captured by the thrombus induced by laser ablation of the endothelial surface of the arterial wall of an artery of the cremaster muscle of the living mouse. The laser wound was administered at 5.5 sec into the record. The platelet thrombus is identified by AlexaFluor 647-labeled anti-CD41 antibody (pseudocolored red). The region of overlap of the green (LPS) and red (CD41) signals is yellow.
(AVI)
